# Sex-specific estimates of positive mental health among youth before and during the COVID-19 pandemic in Canada

**DOI:** 10.24095/hpcdp.45.6.02

**Published:** 2025-06

**Authors:** Florence Lafontaine-Poissant, Laura L. Ooi, Karen C. Roberts, Melanie Varin

**Affiliations:** 1 Centre for Surveillance and Applied Research, Public Health Agency of Canada, Ottawa, Ontario, Canada

**Keywords:** self-rated mental health, life satisfaction, youth, sex differences, COVID-19, Canada

## Abstract

**Introduction:**

Positive mental health (PMH) is an essential component of mental health and well-being. While population-level data show a decrease in youth PMH during the COVID-19 pandemic, there are sex differences that have not been examined.

**Methods:**

Data from the 2017, 2019 and 2021 Canadian Community Health Survey were used to examine youth (12–17 years) PMH before and during the COVID-19 pandemic. Sex-specific prevalence of high self-rated mental health (SRMH) and average life satisfaction (LS) for each year were calculated and disaggregated by sociodemographic characteristics. Differences between years were quantified, and statistical significance was determined using *t* tests (*p* value<0.004 after Bonferroni correction).

**Results:**

From 2019 to 2021, there were significant decreases in the prevalence of high SRMH (from 66.4% to 52.3%) and average LS (8.7 to 8.2) among female youth, at the overall level and across the majority of sociodemographic groups. As for males, no significant decreases were seen at the overall level. After disaggregation, a significant decrease in prevalence of high SRMH was observed from 2019 to 2021 among male youth living in Quebec and nonimmigrant male youth. There were no significant changes in the prevalence of high SRMH or average LS from 2017 to 2019. The sex-specific differences in PMH varied across sociodemographic characteristics.

**Conclusion:**

The PMH of female youth appears to have been affected during the COVID-19 pandemic more than that of male youth. There were sex-specific differences in PMH across sociodemographic groups, suggesting that not all youth were equally affected. Ongoing surveillance with an intersectional lens is needed to better inform public health strategies.

**Highlights**

- Overall, significant decreases in high self-rated mental health (66.4% to 52.3%) and average life satisfaction (8.7 to 8.2) were seen among female youth from 2019 to 2021, but not among male youth.
- There were no changes in high self-rated mental health or average life satisfaction from 2017 to 2019, suggesting that the COVID-19 pandemic and its wider impacts could have contributed to the decrease seen in 2021.
- Significant decreases in high selfrated mental health were seen among males who identified as nonimmigrants, or who lived in Quebec.
- For females, significant decreases in high self-rated mental health and/or mean life satisfaction were seen across every sociodemographic characteristic except ethnic/cultural groups.

## Introduction

Positive mental health (PMH) has been defined by the Public Health Agency of Canada (PHAC) as “the capacity of each and all of us to feel, think, act in ways that enhance our ability to enjoy life and deal with the challenges we face.”^1^ PMH is a priority for Canada,^2^ is a key component of mental health^3^ and can affect physical health.^4^

In 2016, PHAC released the Positive Mental Health Surveillance Indicator Framework (PMHSIF) to monitor PMH and its associated risk and protective factors.^5^ The PMHSIF is a tool that provides a snapshot of the state of PMH among adults (aged 18+ years) and youth (aged 12–17 years) in Canada to inform public health promotion initiatives and policies.^5^,^6^ Monitoring PMH is important from a public health perspective because the skills and attributes associated with PMH can lead to improvement in a range of factors including physical health, preventing the onset of mental health problems, strengthening communities and improving quality of life.^1^

Most of the published literature on PMH has focussed on adults. However, adolescence presents a unique developmental period during which to explore PMH and its determinants. Considerable cognitive, physical, social and emotional changes occur during adolescence,^7^ and youth are often faced with contexts and challenges that differ from those of adults. Although this can make adolescence a period of increased vulnerability to mental health problems,^8^ it can also offer opportunities to establish competencies and experiences that can contribute to well-being.^9^ For example, evidence from a US study shows that youth with PMH were significantly more likely to have stronger psychosocial functioning such as closeness to others and school integration, as well as fewer conduct problems.^10^ Moreover, youth mental health can predict better perceived health and fewer risky health behaviours.^11^ Therefore, surveillance of PMH among youth is important to inform public health guidance, especially in the context of crises such as the COVID-19 pandemic.

To prevent the spread of COVID-19, public health measures (such as physical distancing and school closures) were implemented in Canada, starting in March of 2020. Those measures, along with other challenges stemming from the global pandemic (including health concerns, unemployment, isolation, long COVID, etc.), have affected the Canadian population’s mental health.^12^-^17^ Indeed, PMH outcomes (e.g. high self-rated mental health [SRMH], mean life satisfaction [LS] and high community belonging) decreased from 2020 to 2021 among Canadian adults.^15^,^16^

Similar population-level impacts were seen for youth PMH. In Canada, school closures affected 5.7 million children and youth and resulted in the loss of structure, routine and social connection.^18^-^20^ Those changes may have led to increased worry, helplessness, depression and loneliness.^18^-^20^ Social isolation and reduced protective activities, such as physical activity and seeing friends, could have had numerous detrimental impacts on youths’ mental health.^20^ Indeed, smaller proportions of youth in Canada reported high SRMH in 2021 (62%) compared to 2019 (72%) and 2017 (76%).^6^ In a rapid review, researchers reported that six longitudinal studies indicated a statistically significant decrease in youth well-being and LS during the pandemic compared to the prepandemic period.^21^ Taken together, the COVID-19 pandemic and resulting public health measures appear to have impacted the PMH of youth.

While national-level data indicate that, overall, the PMH of Canadian youth had been decreasing since even before the pandemic,^6^ there are sex differences that should also be taken into account. Specifically, females had a lower prevalence of high SRMH than males both before and during the pandemic.^6^ Additionally, some disparities in mental health outcomes for certain youth populations were seen before the COVID-19 pandemic. For example, students in Grades 6 to 10 with a higher socioeconomic status reported lower LS than students with a lower socioeconomic status.^22^ However, to our knowledge, no study has examined sex differences in youth PMH by sociodemographic subgroup. Filling this data gap is necessary to have a better understanding of possible sex differences in PMH of Canadian youth. Furthermore, understanding how inequities may affect youths’ PMH is crucial for tailoring mental health promotion efforts and developing targeted public education and messaging. 

Accordingly, the objectives of this study were to (1) provide nationally representative, sex-specific estimates of the prevalence of high SRMH and mean LS before (i.e. 2017 and 2019) and during (i.e. 2021) the pandemic; (2) present sex-specific estimates by sociodemographic characteristics to capture intersectional identities; and (3) assess whether the PMH outcomes for males and females, at the national level and across groups, significantly changed across the study period.

## Methods

***Data and participants***

Data were from the 2017, 2019 and 2021 Canadian Community Health Survey (CCHS).^23^-^25^ The CCHS is an annual, national, cross-sectional survey of individuals aged 12 or older in the 10 Canadian provinces and three territories.^23^-^25^ Individuals living on reserves or in other Indigenous settlements in the provinces, full-time members of the Canadian Armed Forces, individuals in institutions and people living in the Quebec health regions of Rgion du Nunavik and Rgion des Terres-Cries-de-la-Baie-James were excluded from this survey’s target population.^23^-^25^ These exclusions represent less than 3% of the population in Canada.^23^-^25^ Survey respondents were selected using a stratified multistage sampling technique.^23^-^25^ Respondents completed the CCHS voluntarily via computer-assisted telephone interview or personal interview.^23^-^25^ In the current study, data were restricted to those aged 12 to 17 years, and limited to those living in the 10 provinces due to the use of single survey years, resulting in a final sample size of 4207 in 2017, 3609 in 2019 and 3283 in 2021.^23^-^25^

***Measures***

**PMH outcomes**

To measure SRMH, participants were asked how they would describe their mental health. Five response options were offered: “excellent,” “very good,” “good,” “fair” and “poor.” Those who answered “excellent” and “very good” were categorized as having high SRMH. To measure LS, participants were asked how they feel about their life right now. Participants answered using a scale from 0 (“very dissatisfied”) to 10 (“very satisfied”). LS was examined as a continuous variable. Both PMH outcomes were based on how they are defined and measured in the youth PMHSIF.^6^

**Sociodemographic variables**

The sociodemographic variables examined in this study were sex (female, male); household income adequacy quintile; region of residence (British Columbia, the Prairies [Alberta, Saskatchewan, Manitoba], the Atlantic provinces [New Brunswick, Prince Edward Island, Nova Scotia, Newfoundland and Labrador], Ontario, Quebec); immigration status (yes, no); place of residence (population centre, rural area); and ethnic/cultural group (Indigenous, Black, East and Southeast Asian, South Asian, Arab/West Asian, Latin American, White). The derived household income quintile variable (INCDVRCA) created by Statistics Canada was modified to report on household income adequacy quintile.^23^-^25^ Place of residence was derived from the participant’s postal code.^23^-^25^ Individuals living in continuously built-up areas with populations of at least 1000 and population densities of at least 400 per km2 were classified as living in population centres.^23^-^25^

***Analysis***

Descriptive statistics were calculated using the Statistical Analysis System (SAS EG) version 7.1 (SAS Institute Inc., Cary, NC, US). Sex-specific prevalence of high SRMH and estimates of mean LS were calculated, overall and disaggregated by sociodemographic characteristics. Estimates were weighted using sampling weights provided by Statistics Canada. We estimated coefficients of variation and 95% confidence intervals (CIs) using bootstrap weights (1000 replicates). Sex-specific estimates of average LS and prevalence of high SRMH in 2017, 2019 and 2021 were compared, with significant differences established using *t* tests. Sex-specific differences within sociodemographic subgroups were established using unadjusted linear or logistic regression. A Bonferroni correction was used to adjust for type 1 error when multiple models were run (e.g. by sex and age group). *P* values were considered stat3istically significant if they were less than 0.004 (0.05/2נ6; we multiplied 2 by 6 because we had 2 sex groups and 6 disaggregates [including overall estimates]). 

## Results

***Sample characteristics***

Sex-specific descriptive statistics are outlined in Table 1. For all three years examined, the majority of the study population lived in Ontario and Quebec, lived in a population centre, were nonimmigrants and identified as White. Across all three years, there were no statistically significant differences across sample characteristics between the male and female populations. 

**Table 1:** Descriptive characteristics, stratified by sex, CCHS 2017, 2019 and 2021

Variables	2017 % (95% CI)		2019 % (95% CI)		2021 % (95% CI)	
	Males	Females	Males	Females	Males	Females
Total unweighted	2156	2051	1836	1773	1703	1580
Total weighted	1 147 311	1 087 876	1 171 489	1 115 051	1 224 882	1 179 889
Household income quintile adequacy						
Q1 (lowest)	24.5 (21.4–27.5)	27.7 (24.6–30.8)	18.2 (15.4–21.1)	21.9 (18.9–24.9)	23.6 (20.2–26.9)	21.3 (18.6–24.0)
Q2	21.4 (18.9–23.9)	18.3 (16.0–20.6)	21.7 (18.9–24.6)	22.0 (19.0–25.0)	19.7 (16.4–23.0)	21.6 (18.5–24.8)
Q3	18.3 (16.1–20.5)	18.2 (16.1–20.4)	23.9 (20.9–26.9)	20.0 (17.3–22.8)	22.3 (19.2–25.4)	22.0 (18.8–25.2)
Q4	20.2 (18.0–22.5)	20.1 (17.6–22.6)	20.5 (17.8–23.2)	21.6 (18.8–24.4)	18.2 (15.5–21.0)	17.9 (15.0–20.7)
Q5 (highest)	15.6 (13.6–17.7)	15.7 (13.5–17.9)	15.6 (13.4–17.8)	14.5 (12.0–17.0)	16.2 (13.7–18.7)	17.2 (14.5–19.8)
Region						
British Columbia	12.7 (12.7–12.7)	12.7 (12.7–12.7)	12.6 (12.6–12.6)	12.5 (12.5–12.5)	12.4 (12.4–12.4)	12.4 (12.4–12.4)
Prairies	19.6 (19.6–19.6)	19.5 (19.5–19.5)	19.8 (19.8–19.8)	19.7 (19.7–19.7)	20.1 (20.0–20.2)	20.2 (20.2–20.3)
Ontario	40.5 (40.5–40.5)	40.5 (40.5–40.5)	40.3 (40.3–40.3)	40.3 (40.3–40.3)	39.8 (39.7–39.8)	39.7 (39.6–39.8)
Quebec	20.9 (20.9–20.9)	21.0 (21.0–21.0)	21.2 (21.2–21.2)	21.3 (21.3–21.3)	21.6 (21.5–21.6)	21.6 (21.6–21.6)
Atlantic	6.3 (6.3–6.3)	6.3 (6.3–6.3)	6.2 (6.2–6.2)	6.2 (6.2–6.2)	6.2 (6.1–6.3)	6.0 (5.9–6.1)
Place of residence						
Population centre	80.6 (78.9–82.3)	81.6 (79.9–83.4)	80.8 (78.9–82.7)	82.1 (80.2–84.0)	80.1 (78.1–82.1)	84.6 (82.9–86.2)
Rural	19.4 (17.7–21.1)	18.4 (16.6–20.1)	19.2 (17.3–21.1)	17.9 (16.0–19.8)	19.9 (17.9–21.9)	15.4 (13.8–17.1)
Immigration status						
Yes	15.4 (13.1–17.8)	15.1 (12.9–17.4)	16.5 (13.8–19.2)	14.3 (11.8–16.9)	16.3 (13.5–19.1)	14.1 (11.3–16.9)
No	84.6 (82.2–86.9)	84.9 (82.6–87.1)	83.5 (80.8–86.2)	85.7 (83.1–88.2)	83.7 (80.9–86.5)	85.9 (83.1–88.7)
Ethnic/cultural group						
White	62.9 (60.2–65.6)	63.3 (60.4–66.3)	63.2 (60.1–66.3)	61.5 (58.3–64.7)	61.2 (57.7–64.7)	61.3 (58.1–64.6)
South Asian	6.8 (5.0–8.5)	7.4 (5.4–9.3)	7.3 (5.2–9.4)	6.9 (5.2–8.6)	6.4^E^ (4.5–8.4)	7.5^E^ (5.2–9.8)
East and Southeast Asian	6.9 (5.4–8.4)	6.3 (4.8–7.7)	12.1 (9.6–14.6)	11.3 (9.0–13.7)	11.1 (8.7–13.5)	9.6 (7.5–11.8)
Black	5.0^E^ (3.2–6.7)	4.7^E^ (2.9–6.5)	5.4 (3.9–6.9)	6.2^E^ (4.2–8.3)	5.2^E^ (3.5–7.0)	6.1^E^ (4.0–8.1)
Arab and West Asian	4.4^E^ (2.7–6.0)	3.6^E^ (2.3–4.8)	2.8^E^ (1.6–4.0)	3.2^E^ (1.9–4.5)	4.4^E^ (2.8–6.1)	5.2^E^ (3.4–6.9)
Latin American	2.2^E^ (1.0–3.4)	1.2^E^ (0.6–1.9)	1.4^E^ (0.7–2.1)	1.6^E^ (0.6–2.6)	3.1^E^ (1.4–4.9)	1.1^E^ (0.4–1.9)
Indigenous	5.9 (4.6–7.3)	5.3 (4.3–6.4)	5.6 (4.3–7.0)	6.5 (5.0–7.9)	5.1 (4.0–6.2)	7.0 (5.3–8.7)

**Abbreviations:** CCHS, Canadian Community Health Survey; CI, confidence interval. 

^E^ Estimate should be interpreted with caution due to high sampling variability (coefficient of variation between 15.0 and 35.0). 

***Sex-specific prevalence estimates of high SRMH before and during the pandemic***

Sex-specific prevalence estimates of high SRMH can be found in Table 2. (The absolute changes for sex-specific prevalence estimates of high self-rated mental health can be found later on, in Table 4.) 

**Table 2:** Sex-specific prevalence estimates of high self-rated mental health among youth aged 12 to 17, disaggregated by sociodemographic characteristic, CCHS 2017, 2019 and 2021

Variables	High self-rated mental health					
	Males % (95% CI)			Females % (95% CI)		
	2017	2019	2021	2017	2019	2021
Overall	79.4 (76.9–81.9)	78.0 (75.0–81.0)	71.8 (68.4–75.2)	72.3^a^ (69.3–75.2)	66.4^a^ (63.0–69.8)	52.3^a^ (48.4–56.3)
Household income quintile adequacy						
Q1 (lowest; ref.)	76.4 (70.4–82.3)	80.2 (74.0–86.5)	66.8 (58.8–74.8)	71.6 (65.2–78.1)	68.5 (61.3–75.7)	55.6 (48.0–63.1)
Q2	81.9 (77.0–86.8)	73.9 (67.0–80.8)	71.4 (62.7–80.1)	75.4 (69.7–81.1)	67.3 (60.0–74.6)	50.5^a^ (41.3–59.6)
Q3	73.8 (67.7–79.8)	75.9 (69.6–82.3)	71.4 (64.4–78.4)	72.3 (66.3–78.2)	62.0 (54.4–69.7)	53.2^a^ (43.7–62.7)
Q4	82.0 (76.8–87.2)	82.0 (75.7–88.4)	74.0 (66.8–81.2)	69.7 (62.9–76.5)	63.3a (56.1–70.5)	52.7^a^ (44.3–61.1)
Q5 (highest)	83.9 (77.8–89.9)	78.8 (72.1–85.6)	77.8 (70.2–85.5)	71.2 (62.7–79.7)	74.5 (67.6–81.3)	51.0^a^ (42.4–59.7)
Region						
British Columbia	80.9 (74.7–87.1)	75.7 (68.5–82.8)	76.9 (69.7–84.1)	72.8 (66.3–79.3)	71.4 (63.6–79.1)	39.9^a^ ^b^ (31.0–48.9)
Prairies	81.8 (77.2–86.4)	75.1 (68.7–81.5)	72.7 (66.3–79.1)	73.1 (67.9–78.3)	64.8 (58.1–71.4)	53.2^a^ (45.9–60.4)
Ontario	79.0 (74.3–83.7)	76.1 (70.4–81.7)	71.3 (64.9–77.8)	68.8 (63.1–74.4)	64.0 (57.6–70.4)	52.1^a^ (44.7–59.4)
Quebec (ref*.*)	78.6 (73.5–83.6)	84.6 (80.5–88.7)	69.2 (62.0–76.4)	76.7 (71.4–81.9)	69.9^a^ (64.2–75.6)	58.5 (51.1–65.8)
Atlantic	73.5 (66.9–80.2)	81.7 (76.1–87.3)	70.6 (62.3–78.9)	75.6 (69.6–81.6)	65.1^a^ (57.3–73.0)	55.4 (45.9–64.8)
Place of residence						
Population centre	79.7 (76.8–82.6)	77.5 (74.0–81.1)	70.6 (66.6–74.7)	72.0a (68.6–75.4)	65.2^a^ (61.2–69.1)	52.0^a^ (47.5–56.5)
Rural (ref*.*)	78.1 (73.3–82.9)	79.9 (75.5–84.3)	76.6 (71.8–81.3)	73.5 (68.6–78.5)	72.2 (66.8–77.6)	54.5^a^ (47.5–61.4)
Immigration status						
Yes	80.9 (74.0–87.8)	77.9 (70.2–85.6)	82.2b (74.4–90.0)	72.3 (63.9–80.7)	76.4 (68.6–84.2)	52.2^a^ (41.6–62.8)
No (ref*.*)	79.1 (76.4–81.8)	78.2 (75.1–81.3)	69.8 (66.1–73.5)	72.3a (69.2–75.3)	64.7^a^ (61.1–68.3)	52.4^a^ (48.3–56.5)
Ethnic/cultural group						
White (ref*.*)	78.8 (75.6–82.0)	77.0 (73.4–80.6)	72.3 (68.0–76.5)	72.2 (68.7–75.6)	66.5^a^ (62.5–70.5)	49.7^a^ (45.1–54.4)
South Asian	82.6 (72.0–93.3)	95.5 (91.3–99.8)	83.7 (72.3–95.1)	67.4 (53.4–81.5)	85.0 (76.2–93.8)	68.0 (52.0–84.0)
East and Southeast Asian	75.5 (65.8–85.1)	74.2 (63.7–84.7)	66.5 (54.7–78.2)	72.8 (61.9–83.6)	68.5 (56.8–80.2)	49.7 (35.3–64.0)
Black	91.0 (81.5–100.5)	82.8 (69.0–96.5)	85.4 (73.7–97.1)	87.5 (76.9–98.1)	67.0 (49.4–84.7)	59.5^E^ (41.1–77.9)
Arab and West Asian	89.2 (78.3–100.0)	77.5 (57.2–97.9)	76.6 (58.7–94.6)	64.1^E^ (43.6–84.5)	79.5 (64.9–94.1)	58.3 (41.8–74.8)
Latin American	74.9^E^ (51.9–97.9)	85.9 (70.5–101.4)	^F^	95.2 (85.5–104.8)	64.3^E^ (33.7–94.9)	57.5^E^ (19.8–95.2)
Indigenous	80.2 (72.5–87.9)	68.7 (56.7–80.7)	59.1 (46.7–71.6)	65.3 (55.1–75.5)	45.3^b^ (33.6–57.0)	44.5 (31.8–57.3)

**Abbreviations**: CCHS, Canadian Community Health Survey; CI, confidence interval; ref., reference group.

^a^ Significantly different compared to males at p < 0.004.

^b^ Significantly different compared to reference group at p < 0.004. 

^E^ Estimate should be interpreted with caution due to high sampling variability (coefficient of variation between 15.0 and 35.0).

^F^ Estimate does not meet Statistics Canada’s quality standard for this statistical program, and should not be published (coefficient of variation above 35.0). 

**Table 4:** Absolute change for sex-specific prevalence estimates of high self-rated mental health and mean life satisfaction estimates among youth aged 12 to 17, disaggregated by sociodemographic characteristics, CCHS 2017, 2019 and 2021

Variables	High self-rated mental health				Mean life satisfaction			
	Males		Females		Males		Females	
	2019 vs. 2017	2021 vs. 2019	2019 vs. 2017	2021 vs. 2019	2019 vs. 2017	2021 vs. 2019	2019 vs. 2017	2021 vs. 2019
Overall	−1.4	−6.2	−5.9	−14.1^*^	−0.1	0.0	0.0	−0.5^*^
Household income quintile adequacy								
Q1 (lowest)	+3.8	−13.4	−3.1	−12.9	−0.1	−0.2	−0.4	−0.1
Q2	−8.0	−2.5	−8.1	−16.8	0.0	+0.1	−0.1	−0.2
Q3	+2.1	−4.5	−10.3	−8.8	+0.1	+0.1	−0.1	−0.5 ^*^
Q4	0.0	−8.0	−6.4	−10.6	0.0	−0.3	+0.3	−0.6 ^*^
Q5 (highest)	−5.1	−1.0	+3.3	−23.5^*^	0.0	−0.1	+0.3	−0.9 ^*^
Region								
British Columbia	−5.2	+1.2	−1.4	−31.5^*^	−0.1	0.0	0.0	−0.7 ^*^
Prairies	−6.7	−2.4	−8.3	−11.6	−0.1	0.0	−0.2	−0.3
Ontario	−2.9	−4.8	−4.8	−11.9	0.0	0.0	0.0	−0.5^*^
Quebec	+6.0	−15.4^*^	−6.8	−11.4	+0.1	−0.3	−0.1	−0.2
Atlantic	+8.2	−11.1	−10.5	−9.7	+0.2	−0.1	0.0	−0.4^*^
Place of residence								
Population centre	−2.2	−6.9	−6.8	−13.2^*^	0.0	−0.1	0.0	−0.5^*^
Rural	+1.8	−3.3	−1.3	−17.7^*^	+0.1	−0.1	−0.1	−0.3
Immigration status								
Yes	−3.0	+4.3	+4.1	−24.2^*^	−0.1	−0.1	0.0	−0.1
No	−0.9	−8.4^*^	−7.6^*^	−12.3^*^	0.0	−0.1	0.0	−0.5^*^
Ethnic/cultural group								
White	−1.8	−4.7	−5.7	−16.8^*^	0.0	−0.1	−0.1	−0.5^*^
South Asian	+12.9	−11.8	+17.6	−17.0	+0.3	0.0	+0.4	−0.2
East and Southeast Asian	−1.3	−7.7	−4.3	−18.8	−0.1	−0.2	−0.1	−0.5
Black	−8.2	+2.6	−20.5	−7.5	−0.1	−0.1	+0.2	−0.5
Arab and West Asian	−11.7	−0.9	+15.4	−21.2	+0.1	−0.3	+0.7	−0.1
Latin American	+11.0	N/A	−30.9	−6.8	0.0	−0.1	−0.4	−1.4
Indigenous	−11.5	−9.6	−20.0	−0.8	−0.2	+0.3	−0.7	−0.3

**Abbreviation**: CCHS, Canadian Community Health Survey. 

^a ^ Bolded estimates indicate a statistically significant difference based on a formal *t* test at *p* < 0.004. 

**Sex differences**

In 2017, 2019 and 2021, females had a significantly lower prevalence of high SRMH compared to males. Across all three years, females living in a population centre and who identified as nonimmigrants had significantly lower prevalences of high SRMH compared to males. In 2019 and 2021, females who were living in the fourth income quintile and who identified as White had significantly lower prevalences of high SRMH compared to males. In 2019, females living in Quebec or the Atlantic provinces had significantly lower prevalences of high SRMH compared to males. In 2021, females in the second, third and fifth income quintiles; females living in British Columbia, Ontario, the Prairies or a rural area; and females who were immigrants reported lower prevalences of high SRMH compared to males. 

***Male youth***

In 2021, prevalence of high SRMH was significantly higher among males who were immigrants (82.2%) compared to those who did not identify as immigrants (69.8%). There were no significant differences in the prevalence of high SRMH by income quintile, region, place of residence, or ethnic/cultural group in either 2017, 2019 or 2021. 

**Changes in prevalence of high SRMH across 2017, 2019 and 2021**

From 2019 to 2021, no significant change in prevalence of high SRMH was observed at the overall level for male youth. However, significant decreases were seen for males living in Quebec (69.2% vs. 84.6%) and those who were nonimmigrants (69.8% vs. 78.2%). There were no significant changes in prevalence of high SRMH from 2017 to 2019.

***Female youth***

In 2019, prevalence of high SRMH was significantly lower among females who identified as Indigenous (45.3%) compared to those who identified as White (66.5%). In 2021, females who lived in British Columbia had a significantly lower prevalence of high SRMH (39.9%) compared to those who lived in Quebec (58.5%). 

**Changes in prevalence of high SRMH across 2017, 2019 and 2021**

For female youth, prevalence of high SRMH was significantly lower in 2021 (52.3%) compared to 2019 (66.4%). In 2021, compared to 2019, there were significant decreases in the prevalence of high SRMH among females in the highest income quintile (51.0% vs. 74.5%); living in British Columbia (39.9% vs. 71.4%); living in a population centre (52.0% vs. 65.2%) or in a rural area (54.5% vs. 72.2%); who were nonimmigrants (52.4% vs. 64.7%) or immigrants (52.2% vs. 76.4%); and who identified as White (49.7% vs. 66.5%). In 2019, prevalence of high SRMH was significantly lower among females who were nonimmigrants (64.7% vs. 72.3%) compared to 2017.

***Sex-specific mean life satisfaction estimates before and during the pandemic***

Sex-specific mean LS estimates can be found in Table 3. The absolute changes for sex-specific average LS estimates can be found in Table 4. 

**Table 3:** Sex-specific mean life satisfaction estimates among youth aged 12 to 17, disaggregated by sociodemographic characteristics, CCHS 2017, 2019 and 2021

Variables	Mean life satisfaction					
	Males Mean (95% CI)			Females Mean (95% CI)		
	2017	2019	2021	2017	2019	2021
Overall	8.8 (8.7–8.8)	8.7 (8.6–8.8)	8.7 (8.6–8.8)	8.7 (8.6–8.8)	8.7 (8.6–8.7)	8.2^a^ (8.1–8.4)
Household income quintile adequacy						
Q1 (lowest; ref.)	8.9 (8.7–9.1)	8.8 (8.6–9.0)	8.6 (8.4–8.8)	8.8 (8.6 –9.0)	8.4 (8.2–8.7)	8.3 (8.0–8.5)
Q2	8.7 (8.6–8.9)	8.7 (8.5–8.9)	8.8 (8.6–9.0)	8.7 (8.5–8.9)	8.6 (8.4–8.8)	8.4 (8.2–8.7)
Q3	8.5b (8.3–8.7)	8.6 (8.4–8.8)	8.7 (8.5–8.9)	8.8 (8.6–8.9)	8.7 (8.5–8.9)	8.2^a^ (7.9–8.4)
Q4	8.8 (8.6–8.9)	8.8 (8.6–9.0)	8.5 (8.3–8.8)	8.5 (8.3–8.7)	8.8^b^ (8.7–9.0)	8.2 (7.9–8.5)
Q5 (highest)	8.8 (8.7–9.0)	8.8 (8.7–9.0)	8.7 (8.5–8.9)	8.6 (8.4–8.9)	8.9^b^ (8.7–9.1)	8.0^a^ (7.7–8.3)
Region						
British Columbia	8.8 (8.5–9.0)	8.7 (8.4–8.9)	8.7 (8.5–8.9)	8.8 (8.6–8.9)	8.8 (8.5–9.0)	8.1^a^ (7.9–8.3)
Prairies	8.8 (8.7–8.9)	8.7 (8.5–8.8)	8.7 (8.5–8.9)	8.8 (8.6–9.0)	8.6 (8.5–8.8)	8.3 (8.1–8.5)
Ontario	8.7 (8.6–8.9)	8.7 (8.5–8.8)	8.7 (8.5–8.8)	8.6 (8.4–8.7)	8.6 (8.5–8.8)	8.1^a^ (7.9–8.3)
Quebec (ref*.*)	8.8 (8.6–8.9)	8.9 (8.8–9.0)	8.6 (8.4–8.8)	8.7 (8.5–8.9)	8.6^a^ (8.5–8.8)	8.4 (8.1–8.7)
Atlantic	8.7 (8.5–8.8)	8.9 (8.7–9.0)	8.8 (8.6–9.0)	8.8 (8.6–8.9)	8.8 (8.6–9.0)	8.4 (8.1–8.6)
Place of residence						
Population centre	8.7 (8.7–8.8)	8.7 (8.6–8.8)	8.6 (8.5–8.7)	8.7 (8.6–8.8)	8.7 (8.6–8.8)	8.2^a^ (8.1–8.3)
Rural (ref*.*)	8.8 (8.7–8.9)	8.9 (8.8–9.1)	8.8 (8.7–9.0)	8.7 (8.6–8.8)	8.6 (8.5–8.8)	8.3^a^ (8.1–8.6)
Immigration status						
Yes	8.9 (8.7–9.0)	8.8 (8.7–9.0)	8.7 (8.5–9.0)	8.6 (8.3–8.9)	8.6 (8.3–8.9)	8.5 (8.1–8.8)
No (ref*.*)	8.7 (8.7–8.8)	8.7 (8.6–8.8)	8.6 (8.5–8.7)	8.7 (8.6–8.8)	8.7 (8.6–8.8)	8.2^a^ (8.1–8.3)
Ethnic/cultural group						
White (ref*.*)	8.7 (8.7–8.8)	8.7 (8.6–8.8)	8.6 (8.5–8.7)	8.7 (8.6–8.8)	8.6 (8.5–8.7)	8.1^a^ (8.0–8.3)
South Asian	8.7 (8.4–9.0)	9.0 (8.7–9.2)	9.0 (8.7–9.3)	8.7 (8.2–9.2)	9.1^b^ (8.8–9.4)	8.9^b^ (8.5–9.3)
East and Southeast Asian	8.6 (8.4–8.9)	8.5 (8.2–8.8)	8.3 (7.8–8.7)	8.9 (8.7–9.2)	8.8 (8.5–9.1)	8.3 (8.0–8.6)
Black	9.2 (8.7–9.6)	9.1 (8.8–9.4)	9.0 (8.7–9.4)	8.4 (7.7–9.0)	8.6 (8.1–9.1)	8.1 (7.5–8.7)
Arab and West Asian	8.8 (8.4–9.1)	8.9 (8.5–9.4)	8.6 (8.1–9.1)	8.4 (7.9–8.9)	9.1 (8.7–9.5)	9.0^b^ (8.6–9.4)
Latin American	8.9 (8.1–9.7)	8.9 (8.3–9.5)	8.8 (8.1–9.6)	9.0 (8.2–9.7)	8.6 (8.0–9.3)	7.2 (5.8–8.7)
Indigenous	8.7 (8.5–8.9)	8.5 (8.1–8.8)	8.8 (8.5–9.0)	8.8 (8.4–9.1)	8.1 (7.6–8.5)	7.8^a^ (7.4–8.2)

**Abbreviations**: CCHS, Canadian Community Health Survey; CI, confidence interval; ref., reference group.

^a^ Significantly different compared to males at *p* < 0.004.

^b^ Significantly different compared to reference group at *p* < 0.004.

**Sex differences**

Although there were no significant differences in average LS between males and females in 2017 or 2019 (overall or across subgroups), average LS was overall significantly lower among females (8.2) compared to males (8.7) in 2021. In 2021, average LS was significantly lower among females compared to males in the third (8.2 vs. 8.7) and fifth (8.0 vs. 8.7) income quintiles; females living in British Columbia or Ontario (8.1 vs. 8.7); females living in a population centre (8.2 vs. 8.6); females living in a rural area (8.3 vs. 8.8); nonimmigrant females (8.2 vs. 8.6); females who identified as White (8.1 vs. 8.6); and females who identified as Indigenous (7.8 vs. 8.8). 

***Male youth***

In 2017, males who were in the third income quintile (8.5) had significantly lower mean LS compared to those in the first income quintile (8.9). Across all three years, there were no significant differences in average LS by region, place of residence, immigration status or ethnic/cultural group. 

***Female youth***

For female youth in 2019, mean LS was significantly higher in the two highest income quintiles (Q4: 8.8, Q5: 8.9) than those in the lowest income quintile (8.4). In 2019 and 2021, mean LS was significantly higher among females who identified as South Asian (9.1 and 8.9, respectively) than those who identified as White (8.6 and 8.1, respectively). In 2021, mean LS was significantly higher among females who identified as Arab and West Asian (9.0) compared to those who identified as White (8.1). Across all three years, there were no significant differences by region, place of residence or immigration status. 

**Changes in mean life satisfaction across 2017, 2019 and 2021**

For both male and female youth, mean LS remained stable from 2017 to 2019 overall and across all sociodemographic groups. There were no significant changes in mean LS from 2019 to 2021 for male youth, overall and across all sociodemographic groups. On the other hand, for female youth, mean LS was significantly lower in 2021 (8.2) compared to 2019 (8.7). From 2019 to 2021, there were significant decreases in mean LS among females in income quintiles Q3 (8.2 vs. 8.7), Q4 (8.2 vs. 8.8) and Q5 (8.0 vs. 8.9); females living in British Columbia (8.1 vs. 8.8), the Atlantic provinces (8.4 vs. 8.8) and Ontario (8.1 vs. 8.6); females living in a population centre (8.2 vs. 8.7); nonimmigrant females (8.2 vs. 8.7); and females who identified as White (8.1 vs. 8.6).

## Discussion

This study examined the sex-specific prevalence of high SRMH and mean LS in Canadian youth across various sociodemographic characteristics in 2017, 2019 and 2021. Consistent with national data,^6^ our findings show that prevalence of high SRMH and mean LS estimates were significantly lower among female youth during the COVID-19 pandemic compared to before. This overall decrease was not seen among male youth. However, there were distinctions in the results after disaggregation. This highlights the importance of (1) incorporating a sex-specific lens, (2) disaggregating the data when possible and (3) exploring different PMH outcomes to better capture the nuanced and potential differential impact of the pandemic on different facets of youths’ PMH. Interestingly, there was no significant change in the two PMH outcomes in 2019 compared to 2017 for either male or female youth overall and across all sociodemographic characteristics. This provides additional evidence for the wider negative impact of the COVID-19 pandemic on youth PMH. Ongoing surveillance is needed to assess whether PMH outcomes return to prepandemic levels.

Among females, there were significant decreases in the prevalence of high SRMH and mean LS across the majority of sociodemographic characteristics. After quantifying the decrease, it was apparent that some populations experienced a larger decrease in PMH than others. For example, the largest decrease in prevalence of high SRMH was seen among females living in British Columbia and the largest decrease in mean LS was among females in the highest income quintile. Among males, the only significant decreases in prevalence of high SRMH were seen among those living in Quebec and nonimmigrants. Targeted mental health promotion activities within these populations may be beneficial. Since this study was descriptive in nature, we cannot identify the reasons for the observed decreases. 

From 2019 to 2021, significant decreases in prevalence of high SRMH and mean LS were primarily observed among female youth. In addition, overall and across the majority of sociodemographic groups, female youth tended to report significantly lower PMH than male youth. These findings add to the extensive body of evidence reporting sex and gender disparities in mental health among youth. 

While sex disparities in PMH had been documented among youth before the COVID-19 pandemic,^26^-^28^ our findings suggest that these inequalities may have increased during the pandemic, thus widening the gender gap.^29^-^31^ Possible explanations for this have been previously published. For example, as social support is a protective factor for PMH,^6^ particularly for women younger than 24 years,^32^ some COVID-19 restrictions such as online school and physical distancing could have impacted female youths’ ability to get the social support they needed,^29^,^30^ which in turn may have had an impact on their well-being. 

Other hypotheses include increased demands placed on females versus males as well as females’ higher levels of anxiety, depression and sadness, changes in sleep patterns, reduced opportunities to meet with friends and lower sense of control over their lives.^30^,^33^,^34^ Future studies examining sex-specific associations between risk or protective factors and PMH are needed to better understand these disparities in the Canadian youth and to tailor targeted public health prevention efforts accordingly.

Unexpectedly, other than among White female youth and nonimmigrants, no other significant changes in PMH were seen across the sociodemographic groups. We suspect that this may be due to smaller sample sizes, which could have reduced the power to detect differences. However, our findings did show Indigenous youth as having the lowest prevalence of high SRMH. This is concerning and highlights the need to find culturally appropriate ways to enhance PMH among Indigenous youth.^35^ There may be macro-level factors, such as race-based discrimination and harassment, that could explain these findings. Indeed, national data from 2020 demonstrate that around half of people who identified as Indigenous reported an increase in discrimination since the start of the COVID-19 pandemic.^36^

The sex-specific results in our study are more nuanced. For example, in 2021, average LS was significantly lower among Indigenous female youth than Indigenous male youth. Factors such as concerns about the impact of stay-at-home orders and concerns about the impact of COVID-19 restrictions on family stress and violence and a lower reported sense of safety in the neighbourhood could have impacted the PMH of female Indigenous youth.^37^ Given that violence, neighbourhood safety and discrimination are risk factors for PMH,^5^,^6^ future studies examining these in more depth along with intersectional identities are needed to form a more comprehensive understanding of PMH among youth. 

***Strengths and limitations***

This study had some noteworthy strengths, including the large sample size in all three years and the national representativeness. This is also the first Canadian study quantifying changes (from 2017 to 2019 and 2019 to 2021) in two PMH outcomes among male and female youth, with an intersectional lens. 

However, there are also some limitations to highlight. As the surveys analyzed were cross-sectional and independent from one another, any changes observed across the three years represent changes at the national level and not at the individual level. The descriptive nature of the analysis did not allow for identification of factors that contributed to any changes in PMH over time. Furthermore, estimates for certain groups could not be released, or were limited, due to insufficient sample sizes. Finally, all of the data in the analysis were self-reported and are therefore subject to reporting and social desirability bias. 

## Conclusion

The PMH of female youth in Canada worsened during the COVID-19 pandemic. This could indicate that existing sex disparities in PMH were exacerbated during that time. Continued surveillance of sex-specific PMH outcomes across various youth subpopulations is needed to help identify groups that may benefit from increased resources and targeted public health prevention strategies. 

## Acknowledgements

The authors would like to thank Statistics Canada for their contribution to the design of the survey, the data collection and the data dissemination. We would like to thank the staff at the Data Coordination and Access Program (DCAP) at the Public Health Agency of Canada for their assistance with data dissemination. Lastly, we would like to thank all of the people who participated in the Canadian Community Health Survey.

## Conflicts of interest

The authors have no conflicts of interest to disclose.

## Authors’ contributions and statement

FLP, LLO, MV: conceptualization.

FLP, MV: formal analysis.

FLP, LLO, MV: methodology.

FLP, MV: project administration.

FLP, MV: writing—original draft.

FLP, LLO, KCR, MV: writing—review and editing. 

The content and views expressed in this article are those of the authors and do not necessarily reflect those of the Government of Canada.
